# Arthropods of Steel Creek, Buffalo National River, Arkansas. III. Heteroptera (Insecta: Hemiptera)

**DOI:** 10.3897/BDJ.4.e7607

**Published:** 2016-05-16

**Authors:** Michael Joseph Skvarla, Danielle M. Fisher, Ashley P.G. Dowling

**Affiliations:** ‡ University of Arkansas, Fayetteville, United States of America

**Keywords:** Heteroptera, Alydidae, Aradidae, Coreidae, Cydnidae, Gerridae, Lygaeidae, Miridae, Pachygronthidae, Pentatomidae, Reduviidae, Rhyparochromidae, Scutelleridae, Thyrecoridae, Tingidae, state record, range expansion, Interior Highlands, Boston Mountains

## Abstract

**Background:**

This is the third in a series of papers detailing the terrestrial arthropods collected during an intensive survey of a site near Steel Creek campground along the Buffalo National River in Arkansas. The survey was conducted over a period of eight and a half months using twelve trap types – Malaise traps, canopy traps (upper and lower collector), Lindgren multifunnel traps (black, green, and purple), pan traps (blue, purple, red, white, and yellow), and pitfall traps – and Berlese-Tullgren extraction of leaf litter.

**New information:**

We provide collection records for 54 species of Heteroptera, 11 of which were new state records for Arkansas: (Aradidae) *Aradus approximatus*, *Aradus duzeei*, *Aradus ornatus*, *Neuroctenus elongatus*, *Neuroctenus pseudonymus*, *Notapictinus aurivilli*; (Cydnidae) *Sehirus cinctus*; (Lygaeidae) *Nysius raphanus*; (Miridae) *Prepops insitivus*; (Reduviidae) *Zelus tetracanthus*; (Rhyparochromidae) *Kolenetrus plenus*.

## Introduction

The Ozarks are a biodiversity hotspot that has been relatively understudied compared to similar areas, such as the Southern Appalachians (Skvarla et al. 2015). This is the third in a series of papers that detail the arthropod fauna collected during a nine month survey conducted in the Boston Mountain subsection of the Ozarks near the Buffalo National River in northwest Arkansas and highlight species newly recorded from the state (for select Coleoptera see Skvarla et al. 2015 and for "Symphyta" see Skvarla et al. 2016). The geologic and biogeographic history of the region and collection methodology of the study were covered in detail by Skvarla et al. 2015.

Recent efforts (e.g., Lee and Barton 1983, Allen and Carlton 1989, Chordas et al. 2005, Chordas and Kremers 2008, Kerzhner and Henry 2008, Scudder 2008, Chordas and Kremers 2009, Taylor and Gill 2009, Henry et al. 2010, Chordas et al. 2011, Swanson 2011, Gaspar et al. 2015) have highlighted the true bug fauna of Arkansas. This paper adds to that effort by reporting 11 new species records of Heteroptera from the state.

## Sampling methods

### Sampling description

The sampling protocol was covered in detail by Skvarla et al. 2015). The following summary is provided for convienience.

The following traps were maintained within the site: five Malaise traps, twenty-five pan traps, fifteen Lindgren multi-funnel traps, four SLAM (Sea, Land, and Air Malaise) traps, and seventeen pitfall trap sets. Additionally, ten leaf litter samples were collected for Berlese extraction when traps were serviced.

All traps were set by 13 March 2013, except Lindgren funnels, which were set on 1 April 2013. Traps set earlier than 13 March were reset on that date in order to standardize trap catch between traps. Traps were serviced approximately every two weeks (14 ±3 days). The last collection of pitfall traps and pan traps occurred on 6 November 2013; Malaise, SLAM, and Lindgren funnel traps were run until 4 December 2013. In total, 1311 samples were collected.

Propylene glycol (in the form of Peak RV & Marine Antifreeze) was used as the preservative in all traps as it is non-toxic and generally preserves specimens well (Skvarla et al. 2014). Insect escape was impeded by the addition of unscented, hypoallergenic detergent to the propylene glycol to act as a surfactant. Trap catch was sieved in the field and stored in Whirl-Pak bags in 90% ethanol until sorting.

### Quality control

Samples were coarse-sorted to easily identifiable levels (generally family, occasionally order or genus) using a Leica MZ16 stereomicroscope illuminated with a Leica KL1500 LCD light source and a Wild M38 stereomicroscope illuminated with an Applied Scientific Devices Corp. Eco-light 20 fiber optic light source. After sorting, specimens were stored in 2 mL microtubes in 70% ethanol.

Specimens were identified with the use of published keys (Table 1). Crasswell 2014) was consulted when the authors were unfamiliar with Heteroptera-specific morphological terms.

## Geographic coverage

### Description

The survey was conducted in 4 hectare plot established at Steel Creek along the Buffalo National River in Newton County, Arkansas, centered at approximately N 36°02.269', W 93°20.434'. For additional details, see Skvarla et al. 2015

### Coordinates

36.0367 and 36.0397 Latitude; -93.3917 and -93.3397 Longitude.

## Usage rights

### Use license

Creative Commons CCZero

## Data resources

### Data package title

Steel Creek survey

### Number of data sets

1

### Data set 1.

#### Data set name

Steel Creek Heteroptera

#### Data format

Darwin Core Archive

#### Number of columns

34

#### Download URL

http://dx.doi.org/10.5061/dryad.b3f33

#### 

**Data set 1.** 

Column label	Column description
typeStatus	Nomenclatural type applied to the record
catalogNumber	Unique within-project and within-lab number applied to the record
recordedBy	Who recorded the record information
individualCount	The number of specimens contained within the record
lifeStage	Life stage of the specimens contained within the record
kingdom	Kingdom name
phylum	Phylum name
class	Class name
order	Order name
family	Family name
genus	Genus name
specificEpithet	Specific epithet
scientificNameAuthorship	Name of the author of the lowest taxon rank included in the record
scientificName	Complete scientific name including author and year
taxonRank	Lowest taxonomic rank of the record
country	Country in which the record was collected
countryCode	Two-letter country code
stateProvince	State in which the record was collected
county	County in which the record was collected
municipality	Closest municipality to where the record was collected
locality	Description of the specific locality where the record was collected
verbatimElevation	Average elevation of the field site in meters
verbatimCoordinates	Approximate center point coordinates of the field site in GPS coordinates
verbatiumLatitude	Approximate center point latitude of the field site in GPS coordinates
verbatimLongitude	Approximate center point longitude of the field site in GPS coordinates
decimalLatitude	Approximate center point latitude of the field site in decimal degrees
decimalLongitude	Approximate center point longitude of the field site in decimal degrees
georeferenceProtocol	Protocol by which the coordinates were taken
identifiedBy	Who identified the record
eventDate	Date or date range the record was collected
habitat	Description of the habitat
language	Two-letter abbreviation of the language in which the data and labels are recorded
institutionCode	Name of the institution where the specimens are deposited
basisofRecord	The specific nature of the record

## Additional information

### Analysis

We collected and identified 672 specimens representing 54 species and 43 genera during this study (Table 1).

Eleven species (20%) represent new state records: (Aradidae) *Aradus approximatus*, *Aradus duzeei*, *Aradus ornatus*, *Neuroctenus elongatus*, *Neuroctenus pseudonymus*, *Notapictinus aurivilli*; (Cydnidae) *Sehirus cinctus*; (Lygaeidae) *Nysius raphanus*; (Miridae) *Prepops insitivus*; (Reduviidae) *Zelus tetracanthus*; (Rhyparochromidae) *Kolenetrus plenus*.

### Notes on selected species

Unless otherwise noted, distribution information was assembled from Kittle 1980, McPherson 1982, Lee and Barton 1983, Henry and Froeschner 1988, Taylor and McPherson 1989, Chordas et al. 2005, Chordas and Kremers 2009, Taylor and Gill 2009, Swanson 2011).

*Aradus approximatus* (Aradidae) is known from Quebec south to Georgia and west to Indiana and Mississippi (Froeschner 1988a). The specimens reported here represent a western range extension.

*Aradus duzeei* (Aradidae) is known from Quebec and Ontario south to Virginia and west to Missouri (Froeschner 1988a).

*Aradus ornatus* (Aradidae) is known from New York and Pennsylvania, south to Georgia, and west to Indiana (Froeschner 1988a). Taylor and Gill 2009 recently reported the species from Louisiana.

*Neuroctenus elongatus* (Aradidae) is known from Pennsylvania, North Carolina, the District of Columbia, Ohio, and Indiana (Froeschner 1988a).

*Neuroctenus pseudonymus* (Aradidae) is known from the District of Columbia, North Carolina, Tennessee, Ohio, Indiana, Texas, and Louisiana (Froeschner 1988a, Taylor and Gill 2009).

*Notapictinus aurivilli* (Aradidae) is known from Florida, Georgia, and Louisiana (Froeschner 1988a, Taylor and Gill 2009). The specimens reported here represent a northern range extension and the first inland records away from Gulf Coastal states.

*Sehirus cinctus* (Cydinidae) is widespread in North America and occurs from Newfoundland and Quebec south to Florida, west to California and south into Mexico (Froeschner 1988b). It has been previously recorded from all states surrounding Arkansas and its occurrence in the state is unsurprising.

*Nysius raphanus* (Lygaeidae) is widespread, being found in North America from Ontario, south to Florida, west to British Columbia, California, and New Mexico; it is also known from Mexico and the West Indies (Ashlock and Slater 1988). It has been previously recorded from Missouri, Kansas, and Texas and its occurrence in Arkansas is unsurprising.

*Preopos insitivus* (Miridae) is widespread in eastern North America, from New Hampshire and Ontario south to Florida, and west to Colorado; it has previously been reported from Missouri (Henry and Wheeler 1988).

*Zelus tetracanthus* (Reduviidae) is widespread in North America and occurs south through Mexico to Paraguay. It has been previously reported from Missouri, Kansas, and Louisiana (Sibley 1951, Swanson 2011).

*Kolenetrus plenus* (Rhyparochromidae) is found in cool, xeric fields transcontinentaly in northern North America from Quebec and Massachusetts west to British Columbia and Yukon; disjunct populations occur in mountainous areas in North Carolina, Montana, Arizona, Mexico, and Guatemala (Slater and Baranowski 1978, Ashlock and Slater 1988, Scudder 1993, Maw et al. 2000). The specimens reported here likely represent a disjunct population that is restricted to the Ozarks or Interior Highlands.

*Xestocoris nitens* (Rhyparochromidae) is known from Quebec, Nova Scotia, and Ontario south to Virginia, west to Michigan, Missouri, and Nebraska (Ashlock and Slater 1988, O'Donnell 2007). Scudder 2010 was the first to record it from Arkansas (Logan County). An unpublished specimen of *X. nitens*, collected in Hempstead County on 5 February 1954 is housed in the University of Arkansas Arthropod Museum and a second unpublished *Xestocoris* from Pulaski County, which is likely *X. nitens*, is housed in Texas A&M University Insect Collection (Quinn 2015).

*Acalypta susanae* (Tingidae) is known from two specimens collected from Mt. Magazine in Arkansas (Allen et al. 1988). The specimens reported here extend the species range northwest into the Boston Mountains and increase the number of specimens in collections. Nymphs, which are undescribed for this species, were collected, although a formal description of immature lifestages is beyond the scope of this work.

### Discussion

The relative abundance of *Acalypta susanae* (33 specimens) collected in this study, when compared to the number of previously known specimens (2), is striking. The species is obviously more widespread than previously thought, but it is unclear without additional sampling effort whether it is locally abundant and the sampled site was particularly good habitat or if they are abundant throughtout their range. As the species is a rather distinctive tingid and easily identified, future leaf litter studies in the Interior Highlands and surrounding area should be observent for additional specimens.

*Kolenetrus plenus* is an interesting species becuase the it has an apparently disjunct range and is restricted to cool, xeric fields in mountainous areas. The specimens reported here are not totally unexpected as the Interior Highlands is the only mountainous region that occurs between the eastern populations in North Carolina and western and southern populations in Arizona and Mexico.

Most of the species newly recorded from Arkansas are widespread in eastern North America and many are known from states that border Arkansas. While their presence in the state is therefore unsurprising, the fact that have have not been previously recorded highlights how under surveyed the state is, especially compared with other biodiversity hotspots.

## Figures and Tables

**Table 1.** Species collected, including total number of specimens. New state records are indicated by an an asterisk (*).

**Family**	**Genus**	**Species**	**To tal specimens collected**	**Identification reference/method**
Alydidae	* Alydus *	*Alydus eurinus*	2	Schaefer 2004 (to genus); one of three *Alydus* in eastern North America, compared to specimens in UAAM (to species)
Alydidae	* Megalotomus *	*Megalotomus quinquespinosus*	45	Schaefer 2004 (to genus); only *Megalotomus* in eastern North America
Aradidae	* Aradus *	*Aradus acutus*	7	Parshley 1921, Blatchley 1926, Usinger and Matsuda 1959, Taylor and Gill 2009)
Aradidae	* Aradus *	Aradus approximatus*	1	Parshley 1921, Blatchley 1926, Usinger and Matsuda 1959, Taylor and Gill 2009)
Aradidae	* Aradus *	*Aradus crenatus*	21	Parshley 1921, Blatchley 1926, Usinger and Matsuda 1959, Taylor and Gill 2009
Aradidae	* Aradus *	Aradus duzeei*	71	Parshley 1921, Blatchley 1926, Usinger and Matsuda 1959, Taylor and Gill 2009
Aradidae	* Aradus *	Aradus ornatus*	3	Parshley 1921, Blatchley 1926, Usinger and Matsuda 1959, Taylor and Gill 2009)
Aradidae	* Aradus *	*Aradus similis*	2	Parshley 1921, Blatchley 1926, Usinger and Matsuda 1959
Aradidae	* Mezira *	*Mezira sayi*	8	Blatchley 1926, Usinger 1936, Usinger and Matsuda 1959, Kormilev 1982b, Davidová-Vilímová et al. 1996, Taylor and Gill 2009)
Aradidae	* Neuroctenus *	Neuroctenus elongatus*	25	Blatchley 1926, Usinger and Matsuda 1959, Kormilev 1982a)
Aradidae	* Neuroctenus *	Neuroctenus pseudonymus*	2	Usinger and Matsuda 1959, Blatchley 1926, Kormilev 1982a, Taylor and Gill 2009
Aradidae	* Notapictinus *	Notapictinus aurivilli*	1	Blatchley 1926, Usinger and Matsuda 1959, Taylor and Gill 2009)
Coreidae	* Acanthocephala *	*Acanthocephala terminalis*	14	distinctive, only species of *Acanthocephala* in Arkansas with brown antenna and contrasting orange terminal segment, no key necessary
Coreidae	* Leptoglossus *	*Leptoglossus oppositus*	10	Baranowski and Slater 1986
Cydnidae	* Amnestus *	*Amnestus basidentatus*	1	McPherson 1982
Cydnidae	* Melanaethus *	*Melanaethus subpunctatus*	2	McPherson 1982
Cydnidae	* Pangaeus *	*Pangaeus bilineatus*	82	McPherson 1982
Cydnidae	* Sehirus *	Sehirus cinctus*	1	McPherson 1982
Gerridae	* Gerris *	*Gerris argenticollis*	1	Kittle 1980)
Gerridae	* Gerris *	*Gerris marginatus*	2	Kittle 1980
Lygaeidae	* Nysius *	Nysius raphanus*	7	Slater and Baranowski 1990
Miridae	* Prepops *	Prepops insitivus*	1	distinctive species, no key necessary
Pachygronthidae	* Oedancala *	*Oedancala dorsalis*	9	Slater and Baranowski 1990)
Pachygronthidae	* Phlegyas *	*Phlegyas abbreviatus*	124	Slater and Baranowski 1990
Pentatomidae	* Banasa *	*Banasa euchlora*	1	McPherson 1982
Pentatomidae	* Brochymena *	*Brochymena arborea*	15	McPherson 1982
Pentatomidae	* Chinavia *	*Chinavia hilaris*	7	McPherson 1982
Pentatomidae	* Euschistus *	*Euschistus servus*	4	McPherson 1982
Pentatomidae	* Euschistus *	*Euschistus tristigmus*	4	McPherson 1982
Pentatomidae	* Hymenarcys *	*Hymenarcys nervosa*	3	McPherson 1982
Pentatomidae	* Menecles *	*Menecles insertus*	22	McPherson 1982
Pentatomidae	* Mormidea *	*Mormidea lugens*	11	McPherson 1982
Pentatomidae	* Podisus *	*Podisus maculiventris*	6	McPherson 1982
Reduviidae	* Arilus *	*Arilus cristatus*	1	distinctive species, no key necessary
Reduviidae	* Barce *		8	not identified to species
Reduviidae	* Melanolestes *	*Melanolestes picipes*	4	distinctive species, no key necessary
Reduviidae	* Oncocephalus *	*Oncocephalus geniculatus*	12	Capriles 1995
Reduviidae	* Pselliopus *	*Pselliopus barberi*	43	Blatchley 1926
Reduviidae	* Rhiginia *	*Rhiginia cruciata*	1	distinctive species, no key necessary
Reduviidae	* Rocconota *	*Rocconota annulicornis*	2	a single species of *Rocconota* occurs north of Mexico
Reduviidae	* Sinea *	*Sinea diadema*	1	Caudell 1901
Reduviidae	* Sinea *	*Sinea spinipes*	5	Caudell 1901
Reduviidae	* Stenopoda *	*Stenopoda spinulosa*	2	Blinn 2012 (to genus); single species of *Stenopoda* north of Mexico
Reduviidae	* Zelus *	Zelus tetracanthus*	1	Hart 1986
Rhyparochromidae	* Antillocoris *	*Antillocoris pilosulus*	1	Slater and Baranowski 1990
Rhyparochromidae	* Cryphula *	*Cryphula trimaculata*	15	Slater and Baranowski 1990
Rhyparochromidae	* Kolenetrus *	Kolenetrus plenus*	3	Slater and Baranowski 1978; a single species of *Kolenetrus*occurs north of Mexico
Rhyparochromidae	* Myodocha *	*Myodocha serripes*	1	distinctive species, no key necessary
Rhyparochromidae	* Ozophora *	*Ozophora picturata*	15	Slater and Baranowski 1990
Rhyparochromidae	* Xestocoris *	*Xestocoris nitens*	1	O'Donnell 2007
Scutelleridae	* Stethaulax *	*Stethaulax marmorata*	4	McPherson 1982)
Thyreocoridae	* Corimelaena *	*Corimelaena pulicaria*	3	McPherson 1982
Thyreocoridae	* Galgupha *	*Galgupha loboprostethia*	4	McPherson 1982
Tingidae	* Acalypta *	*Acalypta susana*	33	Drake and Lattin 1963, Allen et al. 1988)
